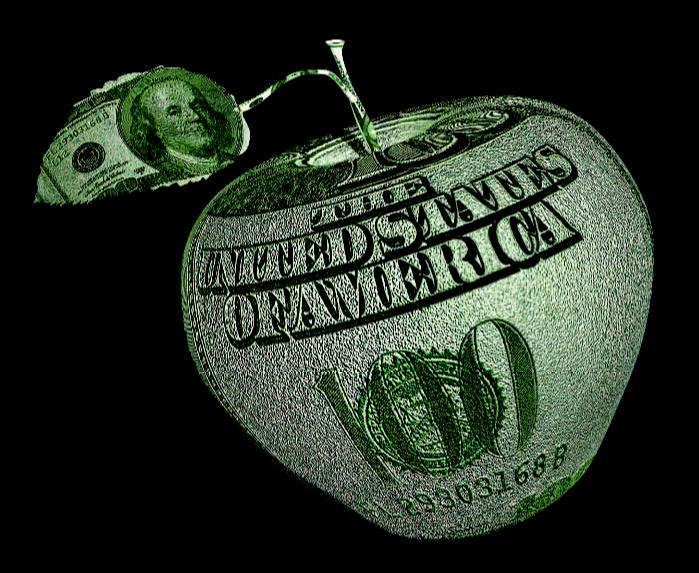# IOM: The Economics of Better Environmental Health

**DOI:** 10.1289/ehp.115-a80

**Published:** 2007-02

**Authors:** Jan Gilbreath

Over the past two decades, epidemiological studies have strengthened the link between air pollution and specific respiratory ailments, yielding better valuations for the pollution-related costs of illness and thus pinpointing the benefits of environmental regulations. Much work remains to be done, however, in linking air pollution to other important health outcomes, such as cancer, infant mortality, and even doctor visits. Nagging questions also remain about how best to translate health effects into economic values.

These were some of the questions addressed at the 14 November 2006 Roundtable on Environmental Health Sciences, Research, and Medicine. a project of the National Academies’ Institute of Medicine cosponsored by the NIEHS along with several other public and private entities. Economists and public health analysts outlined developing methodologies to identify and quantify the health benefits of reduced air pollution and to pinpoint costs to industry of complying with air quality regulations.

“Overall, estimating risk from air pollution is becoming more precise as the pathway from air pollution to health is better characterized,” said C. Arden Pope, a Brigham Young University economist, at the roundtable. Monitoring large groups of people for long periods has enabled researchers to better control for confounding factors such as age, sex, and cigarette smoking, said Pope. More interdisciplinary work is needed to expand the scope of health benefits that can result from reduced pollution as well as further pinpoint measurable compliance costs of regulation.

## Calculating Costs

Health benefits are integrated into regulatory decision making at the end of a complex modeling structure that begins with simulations of air emissions reductions likely to result from a particular regulatory strategy. Other models determine likely changes in human exposure to pollution and probable improvements to public health resulting from the strategy. Improvements to public health—reductions in incidences of disease or mortality—are assigned monetary values so that benefits of regulation can be measured against the costs of implementing and complying with them.

Monetization is controversial largely because of the need to place a value on premature death, and the sheer logistics of the task—simply calculating the number of doctors’ visits for respiratory ailments, for example—can be daunting. Health benefits are calculated either by estimating direct costs associated with avoided illnesses or by assessing the public’s willingness to pay for avoided illnesses.

“Cost of illness” calculations are generally based on hospital admissions and work days lost, which capture direct dollar savings—health care costs avoided by better air quality—but ignore the price of pain and suffering. “Willingness to pay” calculations, based on consumers’ stated or revealed preferences, generally yield less certain results but give a broader picture of total benefits. These calculations give values for premature death, chronic bronchitis, and various respiratory symptoms such as asthma.

Although the cost of premature death traditionally is calculated with the “willingness to pay” method, some international organizations, such as the WHO, instead use a “disability-adjusted life years” or “life years lost” methodology. Within the United States, the use of “life years lost” remains a politically charged debate, said Daniel Greenbaum, president of the Boston-based Health Effects Institute; although “life years lost” calculations may better reflect the impact of air quality regulations, these calculations also routinely lower the estimated benefits of regulation and are criticized as devaluing elderly citizens. This methodology also requires more costly and time-consuming analysis of epidemiological data and a more precise understanding of how the timing of exposure influences health effects.

The EPA’s benefit–cost analyses for regulatory purposes come under strong scrutiny in part because the agency’s assessments of the benefits of EPA regulations exceed those of all other major federal regulations, according to *Progress in Regulatory Reform*, a 2004 report by the Office of Management and Budget. The study calculated the total annual benefits of federal rules from 1993 through 2003 at $63.3–169.3 billion (in 2001 dollars). Of that total, EPA regulations contributed $37.6–131.7 billion in benefits.

The EPA uses costs of lives lost and health effects avoided by reduced pollution in its benefit–cost analyses, Greenbaum said, such as those conducted periodically on the implementation of the Clean Air Act. The EPA has published two *Benefits and Costs of the Clean Air Act* reports to date on this topic. One was a 1970–1990 retrospective study and the other a 1990–2010 prospective study. The latter estimated the value of premature deaths avoided at about $100 billion. Jim DeMocker, senior policy analyst in the EPA Office of Air and Radiation, said the health benefits quantified within this prospective study ranged from $26 billion to $270 billion, depending on the method used to calculate reduced mortality and other benefits.

The EPA is now undertaking a second prospective study of the effects of implementation of the Clean Air Act covering the period 1990–2020, and agency economists are struggling to find the right method for valuing premature death and other health effects. The EPA’s Advisory Council on Clean Air Compliance Analysis has recommended that for this second prospective study, EPA economists revise mortality risk valuation estimates, and estimate exposure and effects of air toxics.

## Toward Better Estimates

Mortality risk valuations are only one set of challenges to benefit–cost analysis. Consensus has not emerged on defining a clear-cut set of health benefits from reduced air pollution or in quantifying the lag time between reductions in exposure to pollution and the realization of health improvements among affected populations. Political controversy continues, as well, on the EPA’s methodologies to determine industry’s costs of regulatory compliance.

Richard D. Morgenstern, senior fellow at the Washington, DC–based Resources for the Future, said extensive surveys of the literature on the EPA’s benefit–cost calculations have shown that both the costs of regulation as well as the potential emission reductions from regulation are overstated, in part because of the difficulties in establishing precise compliance cost estimates before a regulation is implemented. There is less clarity about the accuracy of the EPA’s forecasts on environmental impacts, including health impacts, he said.

Morgenstern identified several cost and benefit factors that are difficult to estimate accurately. One is the impacts associated with technical changes that reduce regulatory compliance costs. “We underestimate technical change,” he said, noting that often “the benefit–cost analysis must reflect the use of certain [proven pollution control] technologies.” In fact, industry often can respond to regulation with more efficient, less expensive technologies that are not easily incorporated into EPA cost calculations. Morgenstern cited a study published in the Spring 2000 issue of the *Journal of Policy Analysis and Management*, which found that, in each of seven cases examined, the EPA’s regulatory impact assessments overestimated the cost of using economic incentives such as emissions trading.

Costs and benefits can be affected as well by changes to prospective rules after benefit–cost calculations are complete, or by incomplete implementation of regulations. Uncertainty associated with calculating baseline air pollution and health conditions before regulations are imposed will also affect how well we can estimate the benefits of new rules.

New frontiers are developing on the scope of health effects linked to air pollution. To date, benefit–cost analyses of air regulations routinely take into account the link between particulate matter pollution and mortality, chronic bronchitis, hospital admissions, asthma-related emergency room visits, acute respiratory symptoms, and asthma attacks. But many other potentially important health effects are not fully quantified or considered, says Greenbaum. Among these are cancer, ozone mortality, infant mortality, decreased lung development in children, doctor visits, and new incidences of asthma. Greenbaum said these health effects are not quantified because researchers lack appropriate baseline incidence rates, because epidemiologists lack enough evidence to link these effects to air pollution, and because the effects are not easily monetized.

As scientists grapple with the uncertainty of estimating and quantifying health benefits of environmental regulation, they are best served by conducting sensitivity analyses of the estimates that they do have, and by stressing post-implementation assessments of benefits and costs, Greenbaum concluded.

Both Greenbaum and Pope also pointed to the need for research that will yield better health care cost estimates. As one example, Greenbaum noted that over time, the prevalence of asthma rises, while treatments for the condition continue to improve—trends that are not captured in benefit–cost analyses of air regulations.

## Figures and Tables

**Figure:**